# Gamma rays impact on 2D-MoS_2_ in water solution

**DOI:** 10.1038/s41598-024-69410-7

**Published:** 2024-09-27

**Authors:** Manjot Singh, Davide Bianco, Jaber Adam, Angela Capaccio, Stefania Clemente, Maria Rosaria Del Sorbo, Chiara Feoli, Jasneet Kaur, Carmela Nappi, Mariarosaria Panico, Giulia Rusciano, Manuela Rossi, Antonio Sasso, Mohammadhassan Valadan, Alberto Cuocolo, Edmondo Battista, Paolo Antonio Netti, Carlo Altucci

**Affiliations:** 1 Department of Advanced Biomedical Sciences, University of Naples, Federico II, Naples, Italy; 2 National Institute of Nuclear Physics, Section of Naples, Naples, Italy; 3 Italian Aerospace Research Centre (CIRA), Capua, Italy; 4 Department of Physics “Ettore Pancini”, University of Naples, Federico II, Naples, Italy; 5 Institute of Biosciences and Bio Resources (IBBR), National Research Council of Italy, Naples, Italy; 6 University Hospital Agency Federico II, Napoli, Italy; 7 Department of Experimental Medicine, University of Campania Luigi Vanvitelli, Naples, Italy; 8 Center for Advanced Biomaterials for HealthCare (CABHC), Italian Institute of Technology, Naples, Italy; 9 Interdisciplinary Research Centre on Biomaterials (CRIB, University of Naples, Federico II, Naples, Italy; 10 Department of Chemical, Materials and Industrial Engineering, University of Naples, Federico II, Naples, Italy; 11 Institute of Biostructure and Bioimaging – CNR, Naples, Italy; 12 Department of Earth Science, Environment and Resources, University of Naples, Federico II, Naples, Italy; 13 Superconducting and Other Innovative Materials and Devices Institute, SPIN-CNR, Naples, Italy; 14 Department of Innovative Technologies in Medicine & Dentistry (DTIMO), University “G. d’Annunzio” Chieti-Pescara, Chieti, Italy; 15 Institute of Applied Sciences and Intelligent Systems, ISASI-CNR, Naples, Italy

**Keywords:** LPE, 2D NSs, Positron annihilation, ^68^Ga irradiation, Binding energy, Deconvolution, Nanoscience and technology, Nanoscale materials, Two-dimensional materials

## Abstract

Two-dimensional transition metal dichalcogenides, particularly MoS_2_, are interesting materials for many applications in aerospace research, radiation therapy and bioscience more in general. Since in many of these applications MoS_2_-based nanomaterials can be placed in an aqueous environment while exposed to ionizing radiation, both experimental and theoretical studies of their behaviour under these conditions is particularly interesting. Here, we study the effects of tiny imparted doses of 511 keV photons to MoS_2_ nanoflakes in water solution. To the best of our knowledge, this is the first study in which ionizing radiation on 2D-MoS_2_ occurs in water. Interestingly, we find that, in addition to the direct interaction between high-energy photons and nanoflakes, reactive chemical species, generated by γ-photons induced radiolysis of water, come into play a relevant role. A radiation transport Monte Carlo simulation allowed determining the elements driving the morphological and spectroscopical changes of 2D-MoS_2_, experimentally monitored by SEM microscopy, DLS, Raman and UV–vis spectroscopy, AFM, and X-ray photoelectron techniques. Our study demonstrates that radiolysis products affect the Molybdenum oxidation state, which is massively changed from the stable + 4 and + 6 states into the rarer and more unstable + 5. These findings will be relevant for radiation-based therapies and diagnostics in patients that are assuming drugs or contrast agents containing 2D-MoS_2_ and for aerospace biomedical applications of 2DMs investigating their actions into living organisms on space station or satellites.

## Introduction

Nanomaterials exhibit noticeable properties strongly related to their fabrication and changes in their bulk counterparts, offering unique physical, chemical, and biological features. Given that, graphene and two-dimensional nanomaterials (2Ds) have gained an increased attention in past decades^1–3^. Within the family of novel nanomaterials, 2D transition metal dichalcogenides (2D-TMDs), such as MoS_2_, WS_2_, MoSe_2_, and WSe_2_, have enticed significant research activities due to the remarkable tunability of their electronic band structure^4,5^, offering a broad range of unique electrical, optical, chemical and mechanical properties^6,7^. In this scenario, 2D-TMDS are considered among the best candidates in various applications^8–10^, including radiotherapy and cancer theranostics through labelling with radionuclides^11–13^ and long-distance quantum communication^14–16^.

The study of the interactions between ionizing radiation and 2DMs has emerged as an important field of research, accounting for the modification in the structure and properties of these novel materials. Impact of radiations on the electrical properties of graphene has been extensively studied, but little is known about their effect on the properties of 2D-TMDs and other novel NMs^17^. One of the most evident consequences of irradiation is the induction of defects, an adverse effect compromising the normal operation of 2DMs-based devices^18–21^.

Herein we present the first study, to the best of our knowledge, of the effect of 2D-MoS_2_ irradiation in water. Irradiation was performed through 511 keV photons resulting from the annihilation of positrons arising in beta decay of ^68^Ga, a radionuclide which has gained attention in the field of oncology and in the clinical application of targeted imaging of various receptors, enzymes, antigens related to diagnosis and discrimination of inflammation and infection diseases^22^.

The motivation for this investigation is evident: the behaviour of nanomaterials in liquid water is intrinsically connected to their interaction in living matter^23–27^. For instance, nano-carriers are used for the delivery of therapeutic radioisotopes into tumours, or of chemotherapeutic drugs for synergistically combined chemo-radiotherapy^28^. The results of our study are also relevant in the field of ionizing radiation dosimetry, where 2DMs and more in general nanocomposites and nanomaterials have found lately massive use to achieve enhanced sensitivity of many dosimetric systems while still achieving tissue equivalency^29^.

A deeper insight on the effect of radiations on 2DMs in a water context surely is greatly impacting on all the possible bio-applications in space, like for example experiments carried on space stations or satellites on biosensing in plants or living organisms or drug delivery to living cells and organisms^30^.

Among the peculiar features resulting from the water-based environment, the production of highly reactive chemical species produced by energetic photons such as hydronium ion, H_3_O^+^, hydroxyl radical and hydroxide surely plays a crucial role^31^. As a matter of fact, the formation of these species is almost absent for irradiation of nanoflakes in air, where it derives from the presence of tiny vapour layers on nanomaterials, as reported in references^18,19^. In addition, the density of the surrounding medium in water is a thousand times higher than in air: this makes the effects of the ionizing radiation treatment on 2DMs quite relevant even at radiation doses much lower than that required to observe analougue impacts in air^32^.

The effect of the irradiation on 2D nanoflakes is studied globally by analysing the shifts of selected Raman features, whereas Atomic Force Microscopy (AFM) is utilized to study the induced structural changes by probing the morphology of the samples. In addition, X-ray Photoelectron Spectroscopy (XPS) is exploited to analyse the generation of defects looking at the significant peak changes in the Mo and S inner shell spectra. The scientific outcomes are supported by a Monte Carlo simulation, describing the interactions between 511 keV photons and MoS_2_ nanoflakes in water environment. In this way, we gained a deeper insight on the physical mechanism for indirect action onto nanoflakes by radical species induced in water by γ-irradiation.

## Results

The aim of the present paper consists indeed in studying the impact of gamma-ray irradiation on 2D-MoS_2_ nanoflakes, produced with a modified liquid-phase exfoliation (LPE) process, in water solution, which represents a novel and very important surrounding context as compared to previous experiments reported for air in the literature.

### Sample production and irradiation

LPE being a very versatile fabrication route gives several opportunities to obtain high quality 2D nanoflakes of varied materials. Compared to other chemical routes of exfoliation, LPE offers an environment friendly and commonly suited synthesis technique with a wide range of solvents resulting in defect free 2D nanoflakes. In the current study, a single step fabrication approach was adopted with a careful optimization of pre- and post-sonication parameters to obtain a stable 2D-MoS_2_ dispersion in liquid water (see Materials and Methods section). Before irradiation, MoS_2_ NSs were subjected to analyse the UV–Vis absorbance spectra in order to extract the basic information on the physical parameters of the exfoliated 2D NSs such as the average lateral size, average final concentration and the average thickness of the 2D NSs (shown in Fig. S1).

Following the fabrication and post fabrication steps, MoS_2_ nanoflakes were subjected to irradiation through 511 keV photons resulting from the annihilation of positrons arising in the beta decay of the ^68^Ga radionuclide.

**Figure 1:**
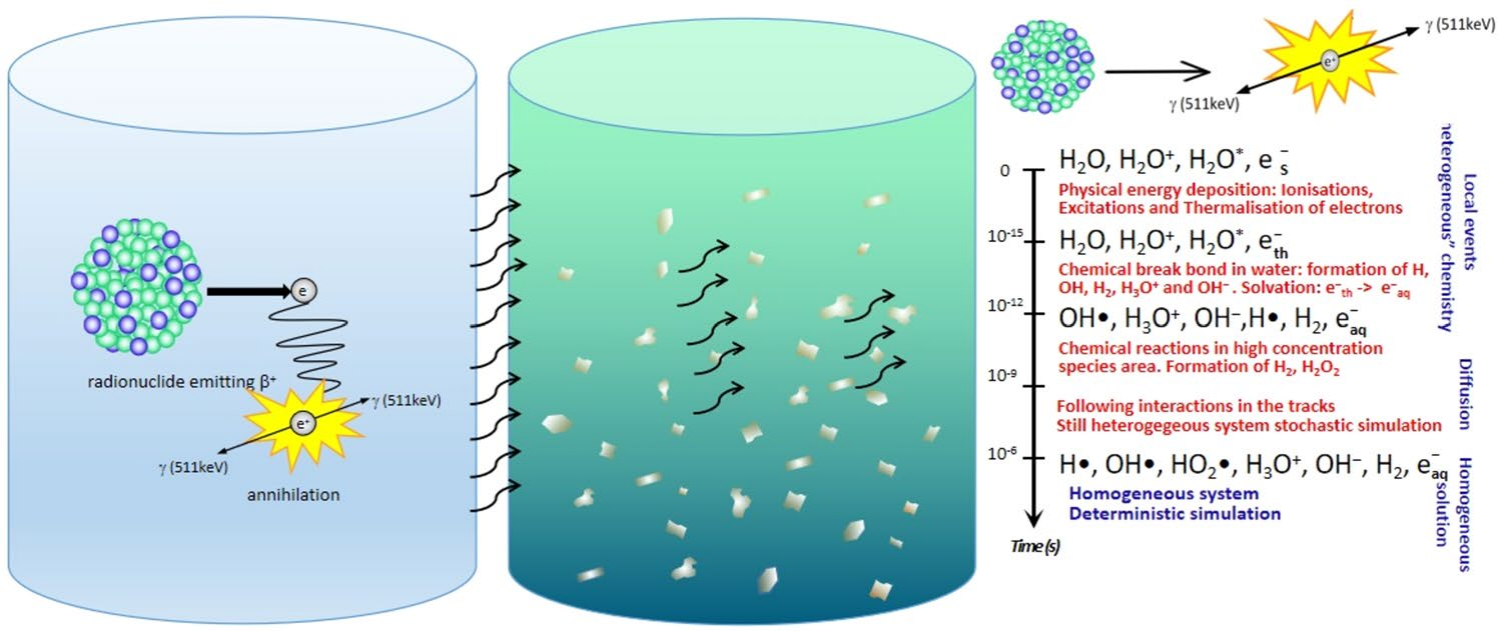
Sketch of the experimental set-up. ^68^Ga solution and 2D-MoS_2_ dispersion were kept separated to study the interactions with produced 511 keV photons and radiolysis products in a water environment.

A scheme of the experimental set-up exploited for irradiating 2D-MoS_2_ dispersion is shown in Fig. 1. A vial containing a pharmaceutical radiolabelled with ^68^Ga, on the left, has been clamped with a vial containing the water solution of 2D-MoS_2_ nanoflakes in a face-to-face configuration. The concentration reached through the exfoliation and centrifugation processes was equal to 50 μg/mL. ^68^Ga decays with a half-life of 67.7 min, through positron emission, to the stable isotope zinc-68 (^68^Zn) with 82–89% yield and an energy spectrum ranging from 242 to 1892 keV^33^.

Water solution determines a fundamentally different irradiation condition with respect to the context^21,32^. Positrons themselves cannot reach the second vial, because of their short range in water and glass. Considering, for instance, the semiempirical approach discussed in^34^, we calculated the mean and maximum positron range in both media by:

1 $${R}_{\text{mean}}(\text{cm})\approx \frac{0.108[{E}_{\beta }^{\text{max}}(\text{MeV}){]}^{1.14}}{\rho ({\text{g cm}}^{-3})},$$

2 $${R}_{\text{max}}\left(\text{cm}\right)\approx \frac{412[{E}_{\beta }^{\text{max}}(\text{MeV}){]}^{n}}{\rho \left(\text{m}{\text{g} \text{cm}}^{-3}\right)}; 0.01\le E\le 2.5 \text{MeV},$$

where the exponent $n$ is given by

3 $$n= 1.265-0.0954\, \text{ln}{E}_{\beta }^{\text{max}}\left(\text{MeV}\right) .$$

Assuming $\rho =1 {\text{g cm}}^{-3}$ for water and $\rho =2.7 {\text{g cm}}^{-3}$ for glass, we obtained the mean and maximum values of the positron range emitted by ^68^Ga, reported in Table S2. In our set-up positrons should travel at least 5 mm of glass before reaching the solution of the second vial containing MoS_2_ nanoflakes, considering the minimum possible crossed thickness of glass (the sum of the wall thickness of the two vials). Therefore, the irradiated vial is only reached by 511 keV photons and lower energy photons originating in Compton scattering, the dominating process in water at this energy.

In order to assess the radiation field experienced by nanoflakes, we performed a simulation of the two-vial experimental set-up with the Geant4 toolkit^35,36^, aiming at determining the characteristics of the secondaries originating from the photon irradiation. In our calculations, we employed at a first glance the low-energy, analytical cross-section derived from the re-engineering of the PENELOPE (PENetration and Energy Loss of Positrons and Electrons) Monte Carlo code^37,38^. In fact, the associated photon interaction model is known to be reliable for photon energies above 100 keV, while allowing a considerable saving of computational time^39^. Consequently, in the first calculation 10^9^ incoming inducing photons were easily employed. A second simulation, giving access to a reliable nanometric representation of the secondaries has been run exploiting Geant4-DNA^36^ and 10^6^ photons.

### Monte Carlo simulation

#### Morphological effects

Following the Monte Carlo simulations of the radiation transport in our experimental set-up, we could figure out the effects of the radioactive ^68^Ga decay on MoS_2_ nanoflakes through the deriving high-energy annihilation photons.

Photons in the considered energy range interact, in water, mainly through Compton scattering. The counterpart to the relatively few, uniformly distributed scattering events originating directly from photons is the interaction pattern of the energetic electrons (delta rays) resulting in ionization events typical of inelastic photon scattering.

**Figure 2:**
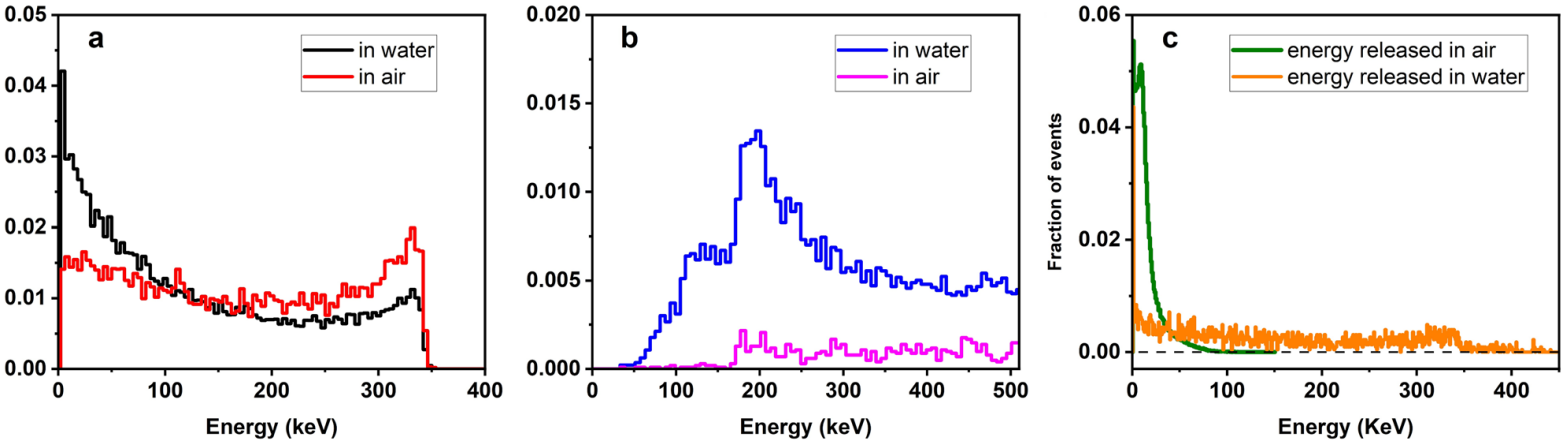
Energy spectrum of electrons ionized by primary photons in air and water medium. (**a**) Energy spectrum of electrons directly ionized by primary photons in water (black), and air (red) originating either in positron annihilation; (**b**) Photon energy spectrum of the Compton scattered component in water (blue) and in air (pink). (**c**) Spectral distributions of energy released in air (black) and water (red) by each interacting photon.

Figure 2 reports for both cases, water and air surrounding environments, the simulated energy spectrum of the electrons directly ionized by photons in the nanoflakes vial, Fig. 2a, whose integral divided by mass is referred to as KERMA (Kinetic Energy Released in Matter) in radiation physics. The energy spectrum of the associated Compton photons is shown in, Fig. 2b, where the contribution of the initial 511 keV photons, representing about half of the available primaries, has not been reported for illustration purposes.

The spectra of Fig. 2 exhibit the typical behaviour of the incoherent Compton scattering, with the characteristic cut-off-like feature of the scattered secondary photon falling at about 1/3 of the primary 511 keV photon energy, 29% in this specific case, regardless the nature of the particular medium, water or air, where the scattering process occurs as predicted by the photon energy differential cross-section for Compton scattering^40^.

A first clear difference between spectra calculated in water and air is the amount of Compton-generated electrons and secondary photons, larger in water with respect to air because of the much higher probability for the interaction of the primary photon to occur in the dense water medium compared to air, according to the interaction probabilities per unit path length ratio, approximately scaling as ${\rho }_{\text{water}}/{\rho }_{\text{air}}$.

A second very clear distinction is that both electron and photon energy spectra of Fig. 2 shows two significantly different shapes: as a matter of fact, the electron energy spectra in water there exhibit a much stronger component in the low energy (< 50 keV) region of the spectrum as compared to air, whereas the secondary photons in water exhibit a consistent shoulder at around 150 keV, which is nearly absent in air. It is worthy noticing that while in a the areas under the black and the red curves are the same since they refer to overall number of secondary electrons emitted in both media, once a single Compton scattering event took place, in b the area under the blue curve in water is much bigger than the area under the pink curve in air since the number of photons that undergo Compton scattering in water is much bigger than in air.

Additionally, in our experiment the radiation interacting with nanoflakes, given their tiny concentration, is almost exclusively composed by secondaries produced in water by the 511 keV primaries and the lower energy photons originated in Compton incoherent scattering.

A quantitative determination of the radiation damage is generally given in terms of macroscopic dose to the irradiated sample. We have calculated the dose imparted to the vial containing the MoS_2_ nanoflakes for comparing with what reported in^32^, where a similar irradiation was performed in air ambient. Exploiting the Monte Carlo calculation a mean energy release per photon in the irradiated vial has been determined as

4 $${\overline{E} }_{\text{evt}} = 3.4\text{ keV}$$

with standard deviation $\sigma$ = 28.8 keV. Considering a total number of decays $N$

5 $$N=\int \frac{dN}{dt}dt=\int A(t) dt= \widetilde{A}={A}_{0} \tau$$

where $A(t)$ indicates the activity at time $t$, ${A}_{0}$ the activity at $t=0$ and $\tau$ the radioactive decay time constant of the Gallium isotope, the mean energy, following all the decays, is given by

6 $$\overline{E }= {\overline{E} }_{evt}\cdot {A}_{0}\cdot \tau .$$

Considering that the vial has a volume $V=\text{1,96} {\text{cm}}^{3}$, the mean energy released corresponds to a mean dose per event ${\overline{D} }_{\text{evt}}=\text{2,78}\cdot {10}^{-13} Gy$, that implies a mean dose, $\overline{D }$, imparted to the nanoflake vial:

7 $$\overline{D }=\text{0,484} Gy.$$

The final imparted dose in air released by the same amount of energy would have been three orders of magnitude smaller, due to the density difference with water.

The differences occurring in the two media can also be understood looking at the distributions in Fig. 2c, where the spectrum of the energy released, respectively, in air and water by each interacting photon has been reported. Photons in water are much more likely to release more energy with respect to air. Photons also have a larger probability of interacting in water, due to its higher density.

Due to the very different mechanism of energy release in the two media, induced interactions with nanoflakes and consequent morphology of the induced damages turns out to be peculiar to the environment medium.

### Nanoscopic analysis—AFM

**Figure 3:**
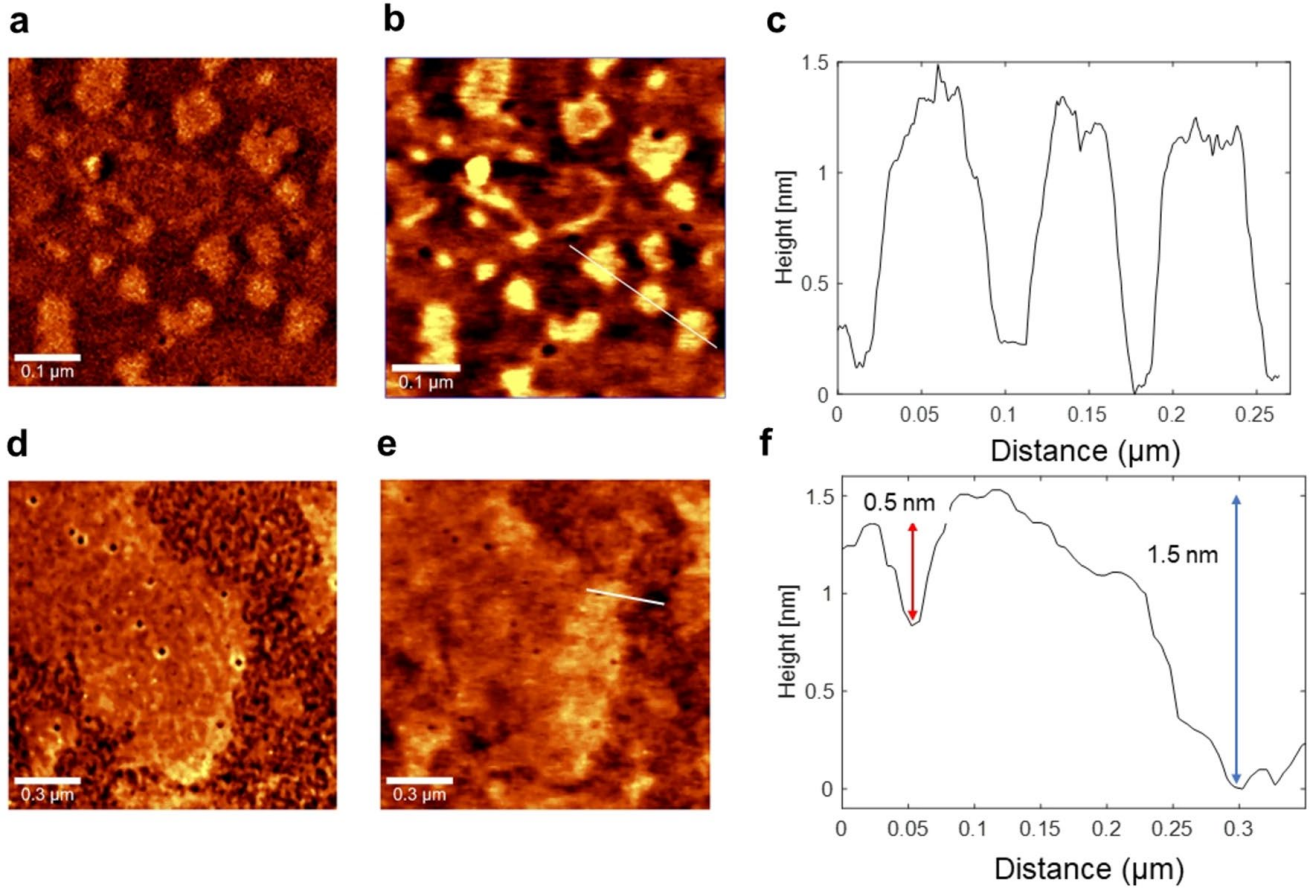
AFM analysis of untreated (**a**–**c**) and treated (**d**-**f**) 2D-MoS_2_ nanoflakes deposited on a Si/SiO_2_ substrate. In particular, panel (**a**, **d**) report typical phase maps, while panel (**b**, **d**) report the corresponding height maps. Finally, panel (**c**, **f**) report the height profiles as read along the white line highlighted in (**b**) and (**e**), respectively.

In order to analyse the nanoflakes morphology and possible modifications induced by direct and/or indirect interactions with ionizing radiations, Atomic Force Microscopy (AFM) was also employed. AFM measurements were performed in AC mode, so that in each scan both phase and morphological (height) maps were acquired. A typical outcome of AFM analysis is shown in Fig. 3 for untreated and treated samples.

The qualitative main features of the acquired maps have been coupled with a quantitative analysis performed over AFM images. As found in AFM images (see Fig. 3d,e)and also in SEM images (Fig. 6 of the following Section), structural damage over the edges, with holes and bulging of the edges, can be clearly appreciated.

**Figure 4:**
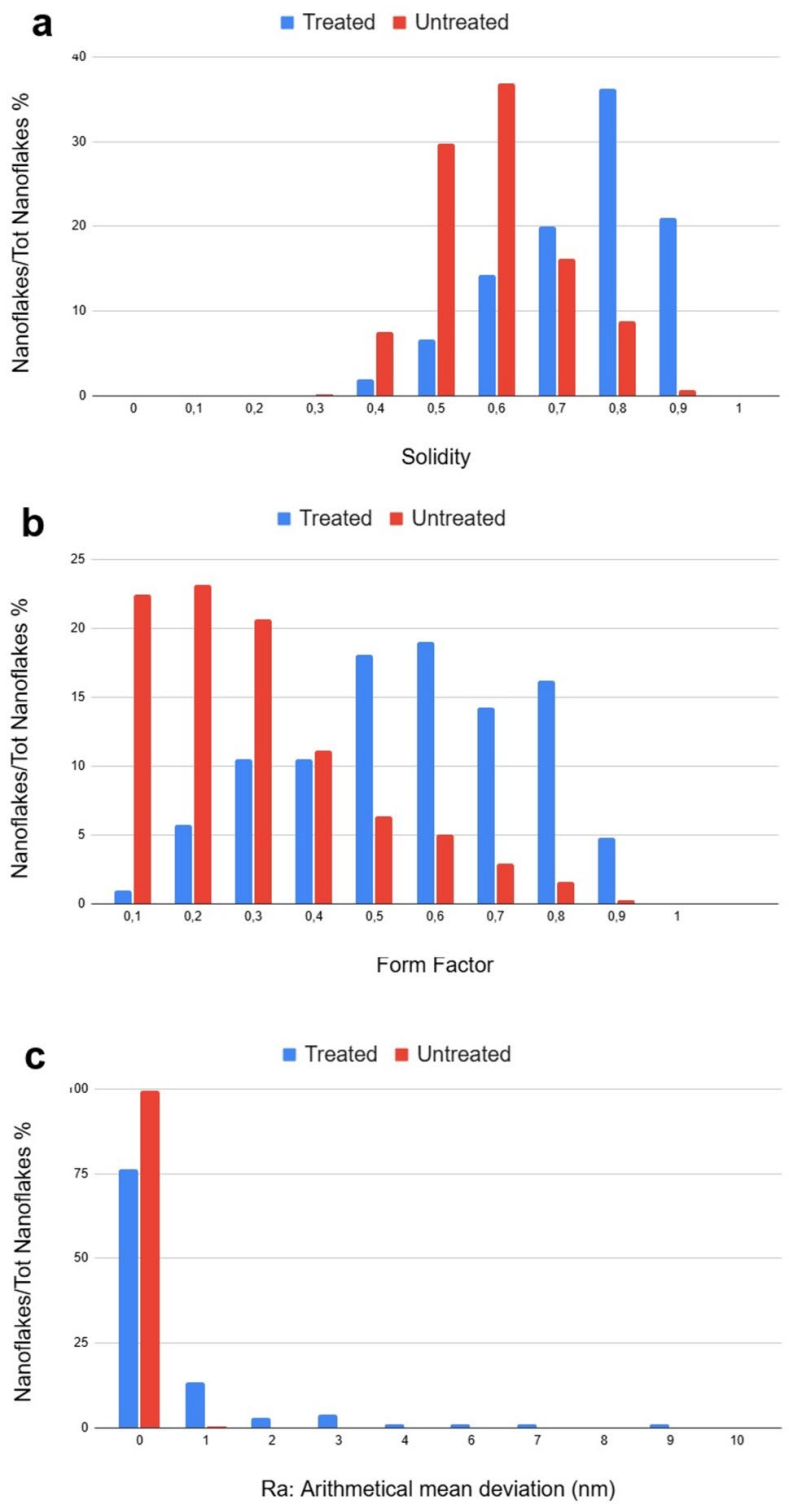
Statistical distribution of (**a**) Solidity and (**b**) Form Factor for untreated and treated cases. (**c**) Histogram graph reports the distribution of the arithmetical mean deviation (average peak-to-valley per nanoflake) Ra values for untreated and treated cases.

In order to point out differences between irradiated and not irradiated samples, a statistical analysis of morphological images has been performed on 2D flakes for each sample. The solidity, namely the ratio of a nanoflake area over its convex hull area, the perimeter, and the area for each nanoflake, automatically segmented with the procedure implemented in ImageJ software package, were extracted. Figure 4 reports the resulting statistical distributions (percentage of the total number of analysed nanoflakes of Solidity (a) and Form Factor (b) are reported, the latter being defined as (4π × Area)/(Perimeter)^2^ and measuring how much the shape of a nanoflake can be defined as circular. As revealed by this figure, nanoflakes Solidity and Form Factor are differently distributed in treated and untreated samples. the value of the main statistical parameters for the measured distributions are reported in Table S3.

Intriguingly, both Solidity and Form Factor increase after irradiation, passing from 0.6 to 0.8 and 0.3 to 0.5, respectively. This suggests a more circular shape and larger convex hull area of the nanoflakes after treatment. This could be explained by the break and removal of the sharpest edges and corners of the nanoflakes because of high-energy radiation treatment at the most likely regions of the nanoflake to be fragmented by ionizing impact.

In order to characterise the impact of ionizing radiation also on the thickness of the 2D-nanoflakes, we have investigated their roughness by analysing the height parameter maps, i.e. the nanoflake thickness, from the AFM topographical images, before and after irradiation. To this purpose, based on the ImageJ software package (Details reported in Methods), we have analysed the statistical variable constituted by the average of the peak-to-valley height per each nanoflake of the probed population, named as the arithmetical mean deviation (Ra), which is a positive variable by definition. Histograms of the measured Ra are plotted in Fig. 4c, for treated and untreated cases, whereas we report in Table S4 the related retrieved statistical parameters. The 100% “0 nm” statistical class of the untreated case indicates that our nanoflake production is fully flat, i.e. nanoflakes have the same thickness over their entire surface, with a 0-nm peak-to-valley value, which is an indication of the high quality of our LPE production resulting in a cut of the nanoflakes along a single crystallographic plane in the production process. Out of this 100% about 75% of the nanoflakes that are treated remains flat with 0-nm Ra value, whereas the residual 25% increases its average peak-to-value because of the impact with ionizing radiations.

Specifically, being the thickness of the MoS_2_ single layer nanoflake ≈ 0.9 nm, we can deduce that most of the induced peak-to-valley excursion is in the range of 1-, 2- or 3-layer thickness. Just a few nanoflakes have a higher Ra value, between 7 and 9 nm, likely due to a possible little aggregation after irradiation, giving rise to a higher Min–Max excursion.

### Microscopic analysis- SEM

**Figure 5:**
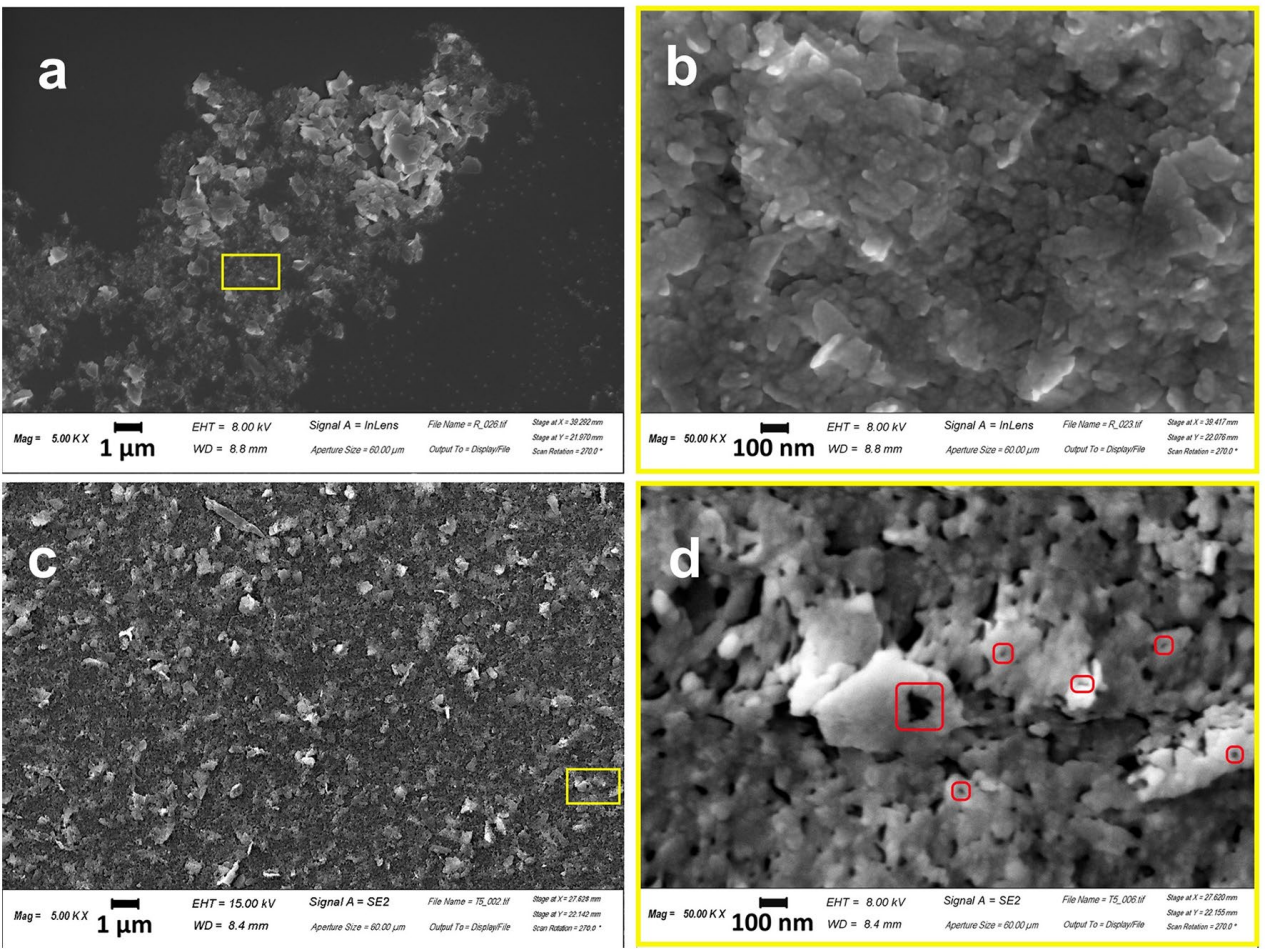
SEM morphological analyses of treated and not treated MoS_2_ NSs. (**a**-**b**) Represents the morphology of non-irradiated but exfoliated MoS_2_ nanoflakes, (**b**) is the magnified (X 10) image of the rectangular area in a, marked by a yellow border and shows a pristine surface of flakes. (**c**-**d**) Represent the irradiated MoS_2_ sample with some structural damage, (**d**) being the magnified (X 10) image of the rectangular area in (**c**), marked by a yellow border. In (**d**) rectangular regions indicated by red borders show details of irradiated samples enlightening the appearance of pores into the nanosheets, induced by radiation treatment.

The impact of irradiation in water onto MoS_2_ nanoflakes morphology has been also analysed by means of Scanning Electron Microscopy (SEM). In Fig. 5a comparison of SEM images of treated and untreated MoS_2_ samples is provided, showing significant differences. Panels of Fig. 5a,b represent the distribution, with an irregular concentration of untreated 2D-MoS_2_ nanoflakes deposited over a silicon substrate, Fig. 5b being the magnified (X 10) image of the rectangular area marked in a by a yellow border. The observed nanoflakes exhibit a typical sharp-edged appearance due to the exfoliation process, distorted rectangular shapes, coherently with the scientific literature^41–43^. The size of the nanoflakes is highly variable, ranging within 33 nm and 1333 nm. Panels of Fig. 5c,d refer to irradiated specimen, where Fig. 5d represents the magnified image (X 10) of the rectangular area marked in Fig. 5c by a yellow border. Two are the main morphological modifications due to irradiation: (i) the appearance of bulged rims together with the disappearance of the sharpest edges in the nanoflake borders and (ii) the appearance of little holes on the nanosheet surface. The first modification implies an increase of the roundness in the flake border shape, in full agreement with the increase in the Form Factor reported in Fig. 4. The second modification implies an increase of the nanoflake mean porosity and would also imply a decrease of the Solidity for the irradiated nanoflakes compared to the non-irradiated case, but its effect is counterbalanced and overtaken by the former modification that, besides the border shape, also affects the Solidity, implying a larger average convex area for the treated nanoflakes, because of the corners removal, in agreement with what observed in Fig. 4. The size of nanosheet is highly variable, ranging within 33 nm and 1000 nm.

Morphology is, somehow, a low-accuracy tool for inspecting damages, since it does not evidence the driver of the changes underwent by the nanoflakes. So, to investigate the chemical nature of the modification induced with the irradiation, we carried out semi-quantitative EDS-WDS chemical analyses on untreated and treated 2D-MoS_2_ nanoflakes. For treated nanoflakes EDS-WDS data indicate, for 15 analysis points out of 20, a higher Mo content together with a minor S content, with a crystal-chemical formula not corresponding to the starting MoS_2_. Five nanoflakes of the analysed sample have regular chemical composition (not altered, in Table S5) whereas fifteen nanoflakes have anomalous chemical composition. Two different reaction mechanism produce the effect of a higher content of Mo together with a lower of S: (i) the molybdenite undergoes a structural modification and a vacancy is created in the S site; (ii) the irradiation causes a chemical alteration of the molybdenite forming MoO_3_ and SO_2_ in an oxidizing atmosphere. Both mechanisms contribute to the observation, since ionization is known to produce atomic vacancies in crystalline structures, as discussed in the following, and the water solvent is mutated into a highly oxidised environment by the irradiation. The stoichiometric effects of both mechanisms on treated nanoflakes are reported in Table S5.

### Spectroscopy findings: Raman spectroscopy

**Figure 6:**
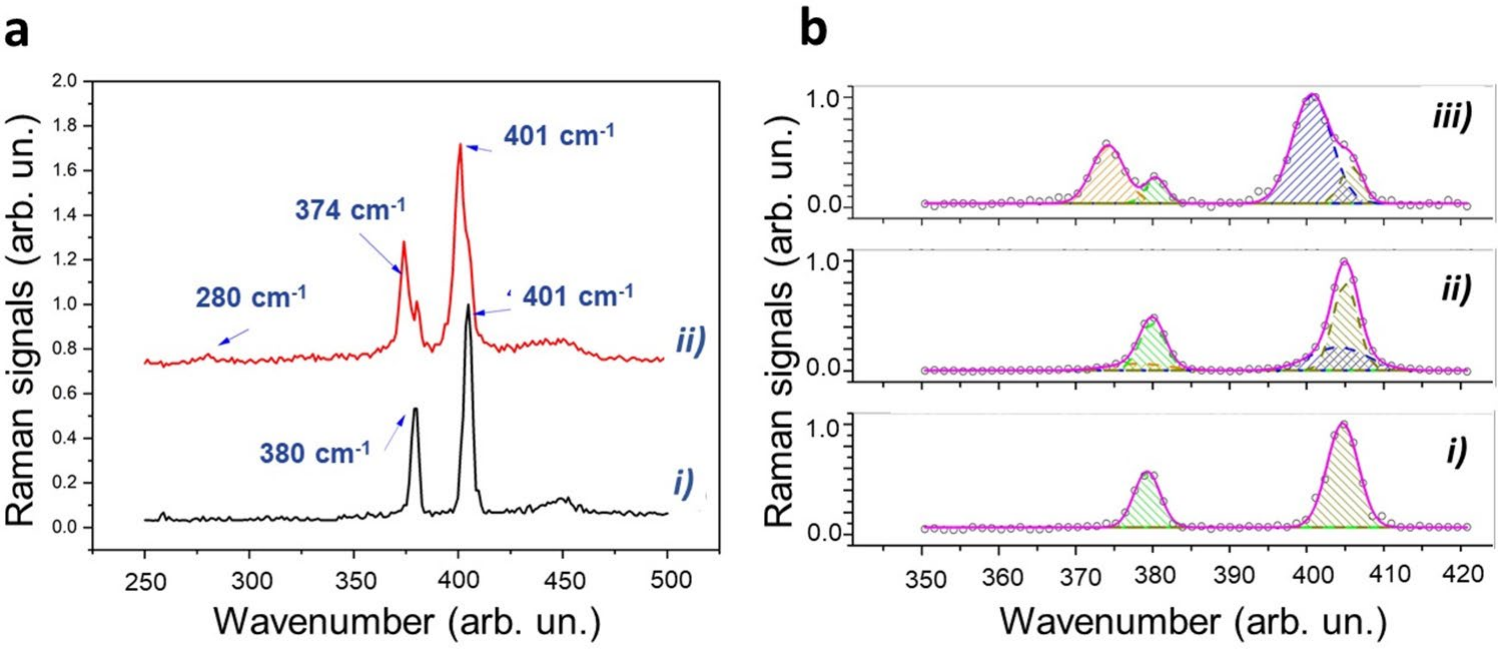
Characteristic Raman spectra of exfoliated treated and not treated MoS_2_ NSs. (**a**) Comparison of selected Raman spectra obtained for not irradiated (trace i) and irradiated (trace ii) flakes. (**b**) Outcomes of the fitting procedure of 2D-MoS_2_ bands in the 350–420 cm^−1^ spectral region. In particular, panel (i) corresponds to the spectrum from untreated flakes, while panel (ii) and (iii) correspond to spectra obtained from irradiated flakes acquired in the inner areas of the flakes (panel ii) and near the border (panel iii). In all the cases, the solid pink lines correspond to the fitted curves, while dashed areas highlight the contribution of the different peaks.

Figure 6 compares selected Raman spectra obtained for untreated (trace *a*) and treated (trace *b*) flakes. Trace* a* clearly exhibits the E^1^_2g_ and A_1g_ modes, which dominates the 250–500 cm^−1^ spectral region of 2D-MoS_2_.

Our Raman analysis revealed that following irradiation, significant changes took place in our nanoflake sample. The most relevant change relies on the apparent splitting of both E^1^_2g_ and A_1g_ modes, resulting by the rise of new, red-shifted peaks. Moreover, the rise of a new feature around 280 cm^−1^, ascribed to the E^1^_g_ mode, can also be appreciated as well as a slight broadening of the band around 450 cm^−1^, due to a combination of a longitudinal acoustic mode (LAM) and an optical mode (A_2u_)^44^. Intriguingly, although such spectral changes exhibit strong point-to-point variations, they are clearly correlated, so that the peak at 280 cm^−1^ is observed when the wider splitting is present. In particular, the most relevant spectral changes are usually found at the flakes (or flake cluster) border, while far from the border only a slight asymmetry of E^1^_2g_ and A_1g_ modes can be seen, what confirms the observation reported in AFM and SEM sections concerning the strong impact of the treatment right on the border of the nanoflakes. To quantitatively describe these changes, Raman spectra corresponding to these conditions were analyzed by a multi-peak fitting in the 350–420 cm^−1^ spectral range. The outcomes of this analysis are shown in Fig. 6b.

Here, panel (i) reports the fitting of the two ${E}_{2g }^{1}\text{and} {A}_{1g}$ bands by two Gaussian curves for of the Raman spectrum corresponding to the untreated sample, while in panels (ii) and (iii) fitting was done with four Gaussian peaks of spectra acquired in irradiated samples at the center (panel ii) and at the border (panel iii). The main features emerging from this analysis can be summarized as follows.

(*) Spectra of irradiated samples can be described as overlapping contributions of unaffected E^1^_2g_ and A_1g_ modes and red-shifted E^1^_2g_ and A_1g_ modes. In particular, a red-shift of 4.2 and 4.0 cm^−1^ was estimated for the E^1^_2g_ and A_1g_ peaks, respectively, for the spectrum in panel (c), while a reduced shift of 1.5 and 1.2 cm^−1^ was estimated for the case in panel (ii);

(**) The FWHM corresponding to shifted modes is more than doubled with respect to unaffected modes;

(***) The relative strength of the two initial peaks is roughly conserved for red-shifted modes.

In order to understand the sample modification induced by $\gamma$-rays irradiation at the basis of the observed spectral changes, some relevant issues have to be highlighted.

First of all, according to literature, both induced strain and doping are mirrored by spectral shift of E^1^_2g_ and A_1g_ modes. In particular, if the changes are induced by strain, the two modes shift in the same direction, with compressive strain causing a blue shift and tensile strain a red shift^45^. On the other hand, doping mainly causes changes for the A_1g_ mode, while E^1^_2g_ mode is much less affected. As a matter of facts, p-doping leads to a blue-shift A_1g_ mode and an increase of its FWHM, while a red shift and a spectral narrowing is associated to n-type doping^46^.

Gamma irradiations of 2D-TMDs has been previously investigated in air for both MoS_2_ and WS_2_ flakes. In the first case, authors observed a red shift of both E^1^_2g_ and A_1g_ modes, which increases with radiation dose. Notably, a ~ 3 cm^−1^ and ~ 3.5 cm^−1^ was observed at a 1000 kGy irradiation dose. Such outcome was ascribed to an increase of interlayers spacing (as confirmed by XRD and TEM analysis), leading to a weakening of the van der Waals interlayer interaction and therefore to a softening of the two Raman modes^47^. On the other hand, it was reported that $\gamma$-irradiation of monolayer WS_2_ leads to a blue shift of the A_1g_ peak and an increase of the intensity ratio between A_1g_ and E^1^_2g_ modes, interpreted in terms creation of vacancies and effective induction of p-doping^46^.

However, more complex and intriguing phenomena can take place for MoS_2_ irradiation in water, due to the presence of $\gamma$-generated ROS species, as discussed in the Conclusions. From our Raman data, although it is not possible to exclude some contributions from doping (e.g. electron doping), it turns out that irradiation leads to a significant weakening of interlayer van der Wall interaction. It is worth noticing that the observed effect is much more pronounced with respect to $\gamma$-irradiation of dry sample reported in^47^. A reasonable cause of that could be related to the intercalation of $\gamma$-generated species, eventually recombining in the interlayer space. It is, in fact, well assessed that ions are able to intercalate into the van der Waals gap through the edges of the 2D flake, causing wrinkling and distortion. Recently, it was also demonstrated that ions can also intercalate through the top surface of few-layer MoS_2_, even reversibly. Previous investigations demonstrated the possibility to check 2D-MoS_2_ intercalation by monitoring the frequency shift of E^1^_2g_ and A_1g_ modes bands. Particularly, it was proved^48^ that O_2_ intercalation in MoS_2_, obtained by a soft-plasma treatment, leads to a weakening of these modes. A red-shift of 0.7 and 0.2 cm^−1^ was observed on average for E^1^_2g_ and A_1g_ bands, respectively, although strong fluctuations are present in the acquired spectra. In that case, authors speculated that oxygen molecules may be formed in the interlayer space after O_2_^+^ ions intercalation, being successively stabilized via the van der Waals interaction with the adjacent MoS_2_ monolayers. A similar intercalation mechanism can likely take place in our experimental condition, where the role of the O_2_^+^ could be played by ions produced in water radiolysis. In this frame, the relevant broadening of red-shifted peaks could reflect the heterogeneity of the process occurring in our case. Clearly, ions interaction with MoS_2_ also introduce a certain amount of structural disorder, that are mirrored by the appearance of the E_1g_ mode (forbidden in back-scattering experiments) and the broadening of the band at 450 cm^−1^.

### X-ray photoelectron spectroscopy (XPS)

**Figure 7:**
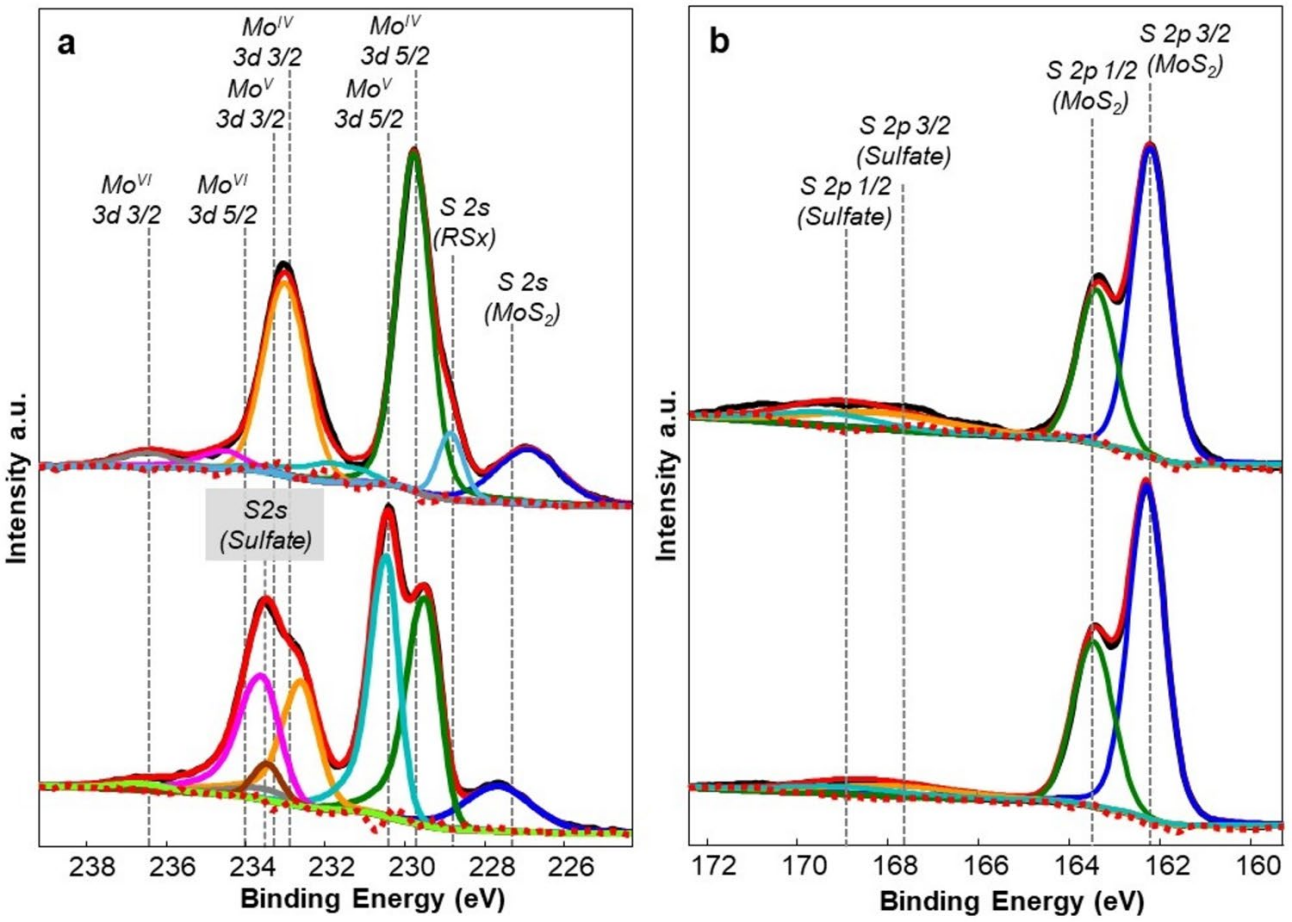
XPS spectra of MoS_2_ exhibiting different oxidized species of Molybdenum and Sulphur atoms. (**a**) Mo 3d band for the untreated case (upper panel), Mo 3d band per the treated case (lower panel), (**b**) S 2p band for the untreated case (upper panel), S 2p band for the treated case (lower panel).

The energy provided to MoS_2_ samples through sonication and subsequent irradiation with γ-photons produces changes in the chemical composition of surfaces as assessed by XPS. In Fig. 7 we report XPS spectra of the Mo 3d band (Fig. 7a, upper and lower graphs for untreated and treated sample, respectively) and the S 2 s band (Fig. 7, upper and lower graphs for untreated and treated sample, respectively). The process of sonication itself, indeed, proved to produce a certain degree of oxidation registered as changes both in the Molybdenum and in the Sulphur spectra^49^.

After deconvolution the Mo 3d spectra of non-irradiated pristine bulk powder consists of one singlet and one doublet in which S 2 s, Mo 3d_5/2_ and Mo 3d_3/2_ photoelectron lines corresponds to Mo^4+^ and S^2-^ environment. The binding energies (BE) of the pristine bulk powder samples are 226.83, 229.51, 232.64 eV^32^ Further values are reported in Table S6 together with the quantitative (percentage) surface composition of samples after deconvolution of the S and Mo envelops, separately normalized into the 159–173 eV—graphs a and b—and 225–239 eV.

As seen in Fig. 7, after irradiation (lower panels) a general important finding is the change in the observed BE, which corresponds to the higher shift in the deconvoluted spectra maintaining Mo^4+^ environment. In case of Mo, the shift in the BE can be attributed to the displacement of S atom because of the direct interaction of incoming γ-photons or the electrons emitted by ionization of the irradiated species which, of course, comes mostly from Compton scattering. Moreover, our experimental setup, in a water environment, implies an enormous amount of oxygen presence resulting in the oxidation of Mo 3d orbitals with a considerable change. The MoS_2_ sample displays the typical peaks of Mo IV, with a characteristic 3d doublet at 229.5 and 232.6 eV, respectively, across all samplings (see Table S6).

The current exfoliation process, which is LPE generates various radical species on the surface of the material induced by sonication as reported in literature^50–52^. In fact, the sonication process introduce (SO_4_)^2-^ resulting in the decrease of the percentage of MoS_2_ from 66.67 to 61.75 as reported in Table S6. The sulfate accounts for 4.91% of the whole composition, which increases after the irradiation to 6.51% (Table S6)^53^. The formation of oxysulfide clusters (Mo_IV_S_y_O_x_) is due to the air contamination and exfoliation process^54^ producing peaks that can be attributed to Molybdenum VI MoO_3_ (above 232 eV and 235 eV). Upon inducing oxygen defects Mo6 + ions are reduced to Mo5 + confirming the formation of MoO_3_^55,56^. Indeed, reducing the size to the nanometric level, during sample fabrication, induces vacancies within the crystal lattice, resulting in edge effects and discontinuities that are more susceptible to oxidation^57,58^. Additionally, species with a BE around 228.47 eV, in Fig. 7a, are attributable to the adsorption of organosulfur molecules grafted by the exfoliation process. These are identified as interfering species that form in materials subjected to high kinetic energy processes^32^.

Regarding the sulfur signals, the S 2p signals of the present MoS_2_ are recognizable as a doublet around 163 eV across all sampling as shown in Fig. 7b while an S 2p fraction of sulfate above 168 eV is recognizable as an effect of oxidation attributable both to the fabrication process and radiolysis. The S 2p spectrum of nonirradiated MoS_2_ consists of a single doublet, in which the S 2p_3/2_ and S 2p_1/2_ photoelectron lines depict the S^2-^ sulfide environment of MoS_2_ as shown in Table S6. The irradiated sample shows another doublet at higher BE in which the S 2p_3/2_ and S 2p_1/2_ photoelectron lines depict the corresponding values at 162.4 and 163.5 eV as shown in Fig. 7b. The reason for this shift at higher BE could be due to the formation of organosulfur species having specific bonds with Oxygen and Carbon atoms that can be ascribed to a decrease in the kinetic energy of the S atoms in the given medium relating it to the formation of bonds with Oxygen and Carbon. The new action mechanism of bond formation with the O and C atoms results in an enhancement of effective nuclear charge over the Mo and S atomic orbitals, whereby reducing electron density in the S atom. The observed BEs for S 2p orbitals are in good agreement with the literature because the thiol and aliphatic sulfide species exhibit BEs in the 162–164 eV range. Interestingly, after irradiation with the given dose, the BE of S 2p orbital increases by few eVs which could be related to the effect of generated radical species such as OH, H_3_O^+^, and H_2_O_2,_ as discussed in the following, facilitating the oxidation of S atoms to produce SO_4_^2−^ species.

As the most striking novel finding resulting from XPS analysis we observe that radiolysis occurring in our experimental setup, produces the massive appearance of the oxidized Molybdenum V fraction. Molybdenum V, which an unstable oxidation state of Molybdenum and is fully absent in the samples before irradiation, exhibits after irradiation a new characteristic big doublet peaked at 230.45 eV—25.37% of the entire surface composition in the Mo spectral band- and at 233.58 eV—17.0% of the entire surface composition- as shown in Fig. 7a. The presence of MoV in previous experiments of Mo nanoflakes exposed in air ambient was much lower and ascribed to the action of residual humidity in air^54^ or to a treatment with an excess of H_2_O_2_. Thus, for the first time to the best of our knowledge the change of the oxidation state of Mo to the unstable MoV into Molybdenum-based nanomaterials is induced by irradiation of samples with γ-photons, the mechanism of which, by the way, substantially agrees with what found in literature^59^, where this is due to the presence of a high concentration of produced radical species such as OH and hydrogen peroxide, due to the action of γ-photons onto the water ambient. Thus, nanoflakes subjected to irradiation present a distinctive subdivision of the Mo fraction into MoIV and MoV species. As also indicated by EDX measurements, the surface is not homogeneous and two distinct populations of MoIV and MoV are evident, while the MoVI fraction becomes less significant. The peak at a BE of 233.45 eV is also noteworthy, which is to attribute to Sulfur 2 s as sulfate, as already reported^60^ likely due to some sulfate ions or oxysulfide clusters remaining, coordinated, or trapped in the lattice of the nanoflakes surface as shown in Fig. 7a.

The atomic surface composition in weight% for pristine MoS_2_, MoS_2_ sonicated, 2D-nanoflakesand treated 2D-nanoflakes is reported in Table S7.

## Discussion

Using the radiochemistry models in Geant4^61,62^, we made some quantitative evaluations concerning the production of species associated with water radiolysis, affecting the nanostructures during and after the so-called “chemical stage” of the irradiation, lasting from 1 ps up to 1 μs after the physical interaction. We calculated the time-dependent radiolytic yields *G*(*t*), defined as the number of molecules produced for a given radiolysis species, at a given time, and normalized to 100 eV of imparted energy:

8 $$G(t)= \frac{N\left(t\right)\cdot 100}{E (eV)}.$$

**Figure 8:**
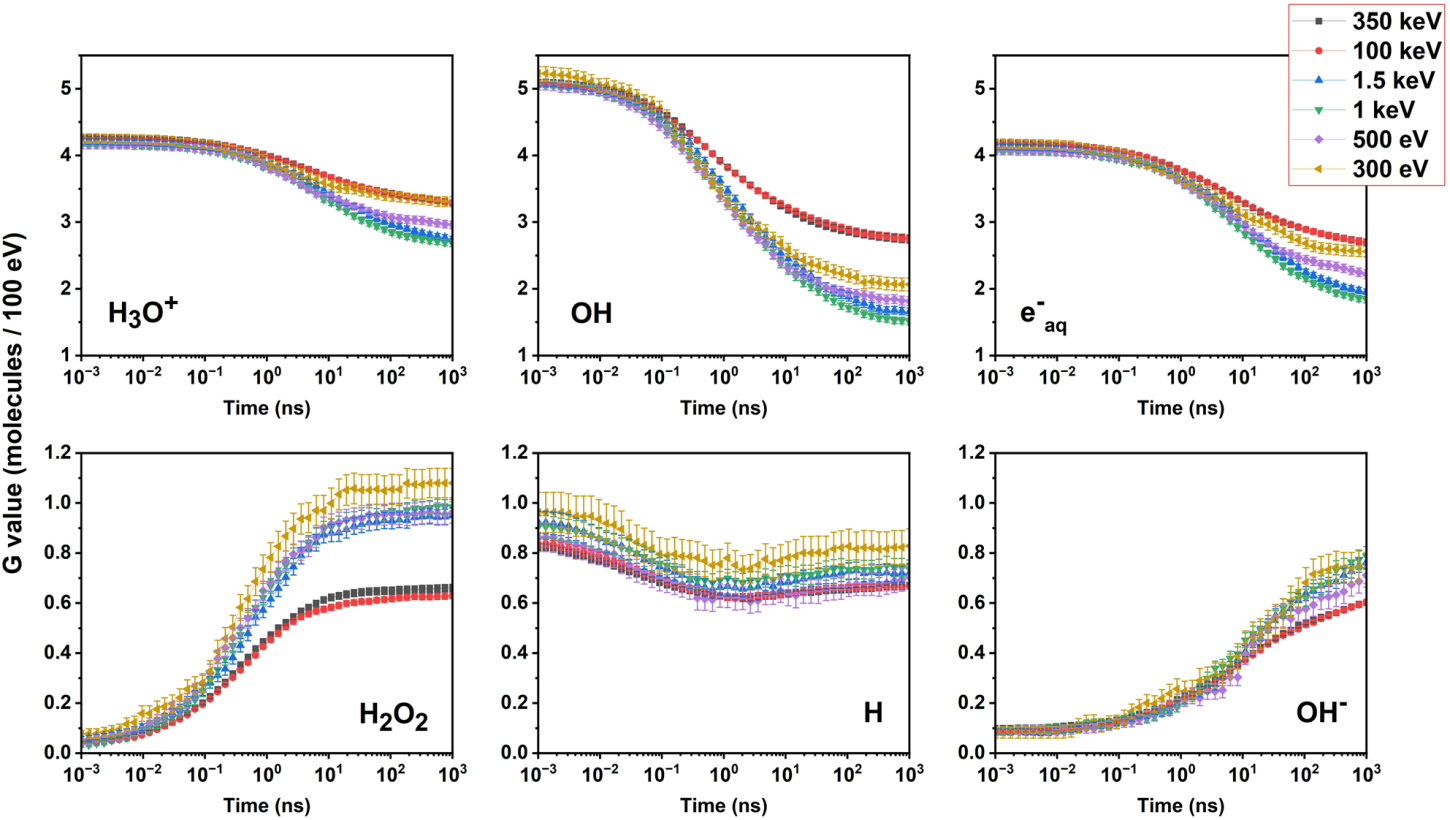
Calculated *G* factor evolution in time at different electron energies for the main hydrolytic species (indicated in each panel), between 1 ps and 1 μs, are reported along with its standard deviation for each calculated point. The Geant4-DNA tool for water radiolysis reported by Shin et al.^64^ has been employed for the calculations.

In Fig. 8, the time evolution between 1 ps and 1 μs of the radiolytic yields is shown for six different species originating in water radiolysis, namely H_3_O^+^, OH, e_aq_, H_2_O_2_, H and OH^-^. Literature dealing with oxidation processes in MoS_2_^63^ suggests that among these the two most active species in changing the chemical nature of the compound adding oxygen could be the hydroxyl radical, OH^-^, extremely reactive and immediately affecting the MoS_2_ nanostructures, and the hydrogen peroxide, H_2_O_2_, more stable although spontaneously dismutating to produce oxygen and water. Then, the indirect effect of the irradiation on the nanoflakes would likely consist of an oxidation of their surfaces.

The most interesting feature to observe in Fig. 8 is the combined evolution of *G*(*t*) for different electron energies for OH and H_2_O_2_, calculated with the Geant4-DNA tool for water radiolysis reported by Shin and coworkers^64^. In fact, while at early times after the radiolysis event, within the first few milliseconds, OH largely prevails due to its much higher G value, at later times the contribution of H_2_O_2_ to the oxidative action can likely become non-negligible due to the increase of its G-value to more than 1 for the generated lower energy electrons in water. This is shown by the behaviour of the green, blue, yellow, and magenta curves in the H_2_O_2_ plot, with energy comprised in the 0.3 – 1.5 keV window. At these later and therefore final stages of the radiolysis process, the H_2_O_2_ G value reached by these lower energy electrons is about double than that reached by the higher energy electrons of the cascade radiolytic process. This implies an important difference in the radiolysis induced by 511 keV γ-photons in water from that induced in air ambient. In fact, in Fig. 2a our calculation shows a much higher production of lower energy electrons in water rather than in air, due to radiolysis of the surrounding medium. These combined calculations imply the production of a very much higher concentration of H_2_O_2_ at later times after radiolysis, as a result of the irradiation in water as compared to air context. The combined action, then, of a massive production of OH at early times and a consistent production of H_2_O_2_ at later times leads in the water environment to specific oxidation mechanisms of MoS_2_ nanoflakes.

## Conclusion

The interactions of gamma photons, originated from positron annihilation, on 2D-MoS_2_ nanoflakes dispersed in water have been carefully and deeply studied. Very interestingly, our findings demonstrate that in addition to the direct interaction between high-energy photons and nanoflakes, the indirect interactions between nanoflakes and radiolysis products generated in water by γ-photons play a fundamental role, which is novel and peculiar to the water environment. The interacting photons induce the generation of various reactive oxygen species in water solution exhibiting a very interesting radiochemistry that affect the MoS_2_ nanoflakes. The key factor for the structural modification of the MoS_2_ nanoflakes is to be found in the species formed in water through radiolysis, that are extremely reactive and can induce oxidation onto the exposed surface of the flakes. In particular, OH radicals are suggested to be dominant in the first few milliseconds after the radiolysis event, due to their high initially produced concentration, whereas H_2_O_2_ could provide a nonnegligible contribution to the oxidation effects at later times, due to its increasing concentration successive to the first few microseconds after radiolysis. As a matter of fact, ionizing radiation in air can similarly induce the formation of reactive species, but their oxidative power is by far smaller than that associated with radicals found in water, for both their resulting concentration and chemical nature. As a novel and intriguing finding, peculiar of the irradiation of samples in water environment, our observations demonstrate that, because of the indirect interactions with the new chemical species formed under water radiolysis, the Molybdenum oxidation state into the nanoflakes is massively changed from the stable + 4 and + 6 state into the rarer and more unstable + 5.

A radiation transport Monte-Carlo simulation, developed for our experimental conditions, allowed determining the main mechanisms driving the morphological and spectroscopical changes of the irradiated nanoflakes.

Raman spectroscopy analysis of the samples confirm the role of oxygenated species, originated by massive water radiolysis. The significant changes observed in the treated samples, giving rise to new red-shifted peaks around 280 cm^−1^ which is ascribed to E^1^_g_ mode, supports the fact that irradiation of 2D-MoS_2_ nanoflakes resulted in significant weakening of interlayer van der Walls interactions. AFM and SEM–EDS analysis clearly showed some structural damage over the edges and significant change in the chemical composition of the Mo and S species. XPS analysis strongly supports and corroborates the whole sample characterization.

A deep morphological and statistical analysis of the treated versus the untreated nanoflakes reveal a more circular shape and larger convex hull area of the nanomaterials after treatment.

Hence, our results open up a very exciting route to explore the effect of high-energy irradiations on the liquid phase exfoliated 2D materials dispersed in water, in view of their intriguing applications in the field of radiotherapy and oncology. Our findings are particularly relevant for very many types of applications of 2D-MoS_2_ from biological and medical studies, where they highlight possible consequences of radiation-based therapies and diagnostics in patients who are assuming drugs or contrast agents containing 2D-MoS_2_, to aero spatial biomedical applications of 2DMs taking place on space station or satellites.

## Methods

### Exfoliation of MoS_2_ powder

The starting commercialized bulk MoS_2_ powder (Sigma Aldrich, 69,860, particle size 6 μm, density 5.06 g mL^−1^ at 25 °C) was exfoliated in pure water as a solvent using a tip sonicator (Bandelin Ultrasound SONOPLUS HD3200, maximum power 200 W, working frequency 20 kHz, KE-76 probe, with an ice-water bath controlling the temperature of the dispersion.

The experimental procedure was adopted from our previous studies but with a little modification^24^. MoS_2_ bulk powder with initial concentration of 5 mg/mL was pre-treated by manual grinding with a pestle-mortar for 50 min. After then, it was exposed to high frequency ultrasonic waves in a tip sonicator and exfoliated for 210 min with a running amplitude of 40% and with pulse mode 10 s On and 10 s Off. Before the sonication step, the immersion of the colloid was done in a broad quartz glass bottle with flat end to maximize the interaction of high sonic jets and bubbles with the stacked bulk MoS_2_ material. Santos et al. has nicely explained the concept of dead zones for an efficient exfoliation which in our case was very helpful in choosing a particular shape of the glass tube for the sonication purpose. The larger the contact area of the probe with the material the more effective the exfoliation and the transfer of acoustic energy and ultrasonic intensity through the probe^65^. The obtained polydisperse 2D-MoS_2_ dispersion was separated into fractions with varying < L > and < N > by controlled centrifugation step. The post sonication step, which is the liquid cascade centrifugation, was employed to obtain the 2D nanosheets with controlled size and thickness^23,65^. Successive stepwise controlled centrifugation steps were carried out (Megafuge 16 centrifuge, a swinging bucket rotor centrifuge device) at 100 g and 1000 g for 60 min each to analyse the supernatants. The as obtained 2D MoS_2_ NSs was first centrifuged at 100 g for 60 min and the resulting sediment was discarded because of the mostly unexfoliated material and the supernatant is shifted to higher centrifuge step of 1000 g for 60 min. After this step, the final supernatant was stored at 4 °C, used for further characterizations and for the Irradiation purpose.

### Description of the vial with ^68^Ga radiation source preparation

^68^Ga-chloride has been eluted directly from a germanium-68 (^68^Ge) /gallium-68 (^68^Ga) generator (Eckert and Ziegler Radio pharma GmbH) with a 270.93-day half-life of the parent nuclide ^68^Ge. The ^68^Ge/^68^Ga generator is designed to elute all of the available ^68^Ga activity in a vial with a volume of 5 ml of sterile ultrapure 0.1 mol/l hydrochloric acid. Eluted activity has been measured right after elution with Capintec™ Dose Calibrator.

### Monte Carlo simulation

GEANT4, short for Geometry and Tracking 4, is a freely available Monte Carlo Simulation toolkit developed in C +  + with an object-oriented approach^35,36^. The Geant4 version that has been used for the simulations in our paper is 11.2. The link for downloading the latest version of the software is the following: https://geant4.web.cern.ch/. It originated from the collaborative efforts of over 100 researchers under the RD44 collaboration. In this study, we employed the toolkit to simulate the two-vial experimental set-up of the MoS_2_ NSs. The geometry of the system was modelled assuming a distribution of nanometric flakes in line with the concentration employed. At a first stage the interaction of the 511keV and Compton photons with the nanoflakes vial has been reproduced employing the low-energy, analytical cross-section derived from the re-engineering of the PENELOPE (PENetration and Energy Loss of Positrons and Electrons) Monte Carlo code^37,38^ that allows a considerable saving of computational time while remaining reliable for photons originating in the vial with the radioactive Gadolinium from the positron annihilation. Subsequently, to access the track structure of secondary electrons, another simulation campaign has been carried on with the Geant4-DNA^36^ package and 10^6^ photons. The Geant4-DNA project was initially launched by the European Space Agency to study the biological effects of radiation during extended manned space missions. The associated software packages include the refinement of electromagnetic physics models to accurately represent interactions down to the eV energy scale, and the integration of water radiolysis processes to account for the generation of oxidative radical species, in order to simulate both direct and non-direct ionizing radiation biological effects at the DNA level in nanodosimetric studies^66^.

Using the radiochemistry models in Geant4-DNA^61,62^ we calculated yields of different species deriving from water radiolysis during and after the so-called “chemical stage” of the irradiation, lasting from 1 ps up to 1 μs after the physical interaction. To this end, the irradiation of a millimetric water cube with a scaled flakes distribution has been simulated, employing electrons with energies ranging from 300 eV to 350 keV. The resulting time-dependent radiolytic yields *G*(*t*), representing the number of molecules produced for a given radiolysis species normalized in energy, has been calculated for H_3_O^+^, OH, e_aq_, H_2_O_2_, H and OH^-^.

## Supplementary Information

Supplementary Information.

## Data Availability

Availability of Data and Materials The datasets used and/or analysed during the current study are available from the corresponding authors on reasonable request.
