# Supplementary material for: Gamma rays impact on 2D-MoS2 in water solution
Source: Sci Rep. 2024 Sep 27;14:22130. doi: 10.1038/s41598-024-69410-7 (PMC11437032; doi:10.1038/s41598-024-69410-7)
Supplement: Supplementary file 1 — Supplementary Information. [file 41598_2024_69410_MOESM1_ESM.pdf]

Gamma rays impact on 2D-MoS_2 in water solution

Manjot Singh[1,2,§], Davide Bianco[1,2,3,§], Jaber Adam[5], Angela Capaccio[4,5], Stefania Clemente[6], Maria Rosaria Del Sorbo[7], Chiara Feoli[1], Jasneet Kaur[5], Carmela Nappi[1], Mariarosaria Panico[11], Giulia Rusciano[5], Manuela Rossi[12], Antonio Sasso[5], Mohammadhassan Valadan[1,2,13], Alberto Cuocolo[1,*] Edmondo Battista[8,14,*], Paolo Antonio Netti[8,9,10*], and Carlo Altucci[1,2,15*]

[1]Department of Advanced Biomedical Sciences, University of Naples, Federico II (ITALY)

[2]National Institute of Nuclear Physics, Section of Naples (ITALY)

[3]Italian Aerospace Research Centre (CIRA), Capua (ITALY)

[4]Institute of Biosciences and Bio Resources (IBBR), National Research Council of Italy, Naples (ITALY)

[5]Department of Physics "Ettore Pancini", University of Naples, Federico II (ITALY)

[6]University Hospital Agency Federico II, Napoli (ITALY)

[7]Department of experimental medicine, University of Campania Luigi Vanvitelli, Naples (ITALY)

[8]Center for Advanced Biomaterials for HealthCare (CABHC), Italian Institute of Technology, Naples (ITALY)

[9]Interdisciplinary Research Centre on Biomaterials (CRIB) University of Naples, Federico II (ITALY)

[10]Department of Chemical, Materials and Industrial Engineering, University of Naples, Federico II (ITALY)

[11]Institute of Biostructure and Bioimaging – CNR, Naples (ITALY)

[12]Department of Earth Science, Environment and Resources, University of Naples, Federico II (ITALY)

[13]Superconducting and other Innovative materials and devices Institute, SPIN-CNR, Naples (ITALY)

[14]Department of Innovative Technologies in Medicine & Dentistry (DTIMO), University "G. d'Annunzio" Chieti-Pescara, Chieti, (ITALY))

[15]Institute of Applied Sciences and Intelligent Systems, ISASI-CNR, Naples (ITALY)

§These authors equally contributed

[*]Corresponding author

Sample characterization

UV-Visible absorbance Spectroscopy

The basic information about the physical parameters of the exfoliated 2D NSs, such as the < N >, < L >, and Mean concentration < L >, were extracted from UV-VIS extinction spectra acquired by a V-730 Jasco UV-Visible Spectrophotometer. The extinction spectra contain the contribution from both absorbance and scattering components. We quantified the physical parameters of 2D NSs from these two components using formula reported in literature[1,2] our experimental protocol, at lower centrifugal force 100 g, scattering component was more dominant with high extinction peaks at 680 nm, so we discarded this sample for any further characterization. Whereas, at 1000 g, the two exciton peaks, A-exciton, and B- exciton shifted towards lower wavelength region and consequently few-layered enriched dispersion is obtained.

The extinction spectra of MoS_2 after the final step of centrifugation at 1000 g is shown in

Figure S1. After irradiating the sample, a red shift in the two excitonic peaks is observed which corresponds to the change in the layer number and lateral size of the irradiated treated 2D NSs.

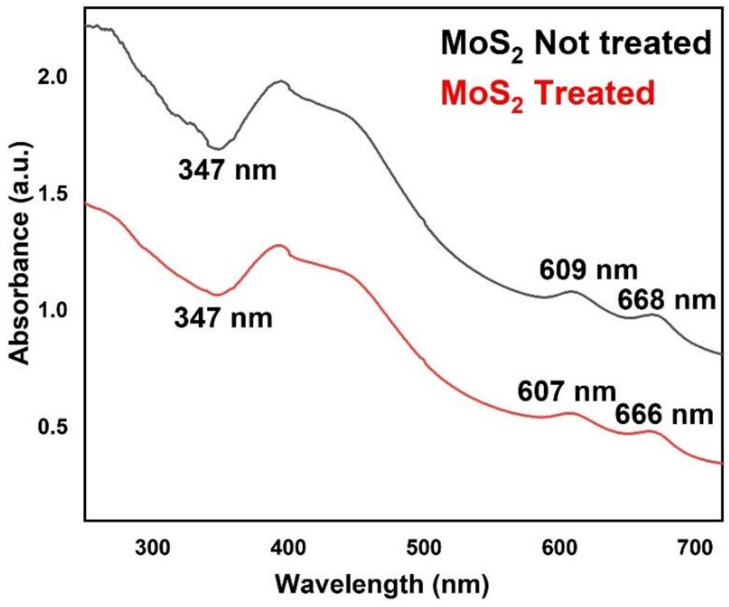

Figure S1: Corresponds to the comparative UV-vis absorbance spectra of Not treated MoS_2 dispersion and irradiated-treated 2D MoS_2 dispersion with ^{68}Ga radioisotope photons, respectively.

Dynamic Light Scattering (DLS): size and ζ-Potential measurements

Size measurements of 2D-MoS_2 were performed by DLS technique using a Malvern Zetasizer nano ZS. Data reported is taken as the average of three measurements (n = 3) measured at 25 °C in disposable folded capillary cells (DTS1070) in water dispersants.

Liquid phase exfoliation produces also surface charges of the 2D-MoS_2 NSs which plays an important role in understanding the stability kinetics of liquid exfoliated dispersions. At this purpose ζ-Potential measurements were also performed. These measurements were carried out on laser interferometric technique (Malvern Zetasizer Nano system) with irradiation from 633 nm He-Ne laser. The samples were injected in folded capillary cells, and the electrophoretic mobility (μ) value was measured using a combination of electrophoresis and laser Doppler velocimetry techniques. All the measurements were carried out at 25°C.

Surface charge present on the exfoliated 2D NSs is essential in deciding its fate in its interaction with the outer context. The quantitative information obtained from the measured electrophoretic mobility and surface charge in a given medium explains the stability kinetics of exfoliated 2D NSs, in pure water or in any given solvent. We observed a nearly unchanged NS ζ-Potential after radiation treatment with a tiny decrease of its negative value and, thus, a small decrease in the negative overall charge value accumulated over the NS surfaces. This, in turn, implies also a little decrease in the stability of the dispersion in water over the two weeks. The slight decrease in the ζ-Potential negative values could be attributed to a moderate partialization of the initial negative surface charges with some H^+ ions present in solution because of the well-known slightly acidic behavior of the H_2O_2 excess, produced by radiolysis induced by the ionizing radiation interaction with water solvent. Moreover, the little decrease trend remained quite consistent in all the software runs over the measurement duration.

Also, mean size of exfoliated 2D NSs measured by DLS showed a trend to aggregation of the nanoflakes after treatment. In fact, the average DLS value measured for exfoliated untreated 2D NSs was ≈ 190 nm, whereas after irradiation, the average size of 2D MoS_2 NSs increased to about 220 nm, as shown in Table S1. These findings explain why the treatment of 2D NSs with γ-photons in water solution affects the stability kinetics of the NSs. The statistical value of the mean DLS size and ζ-Potential for the probed nanoflakes is reported in Table S1.

2D NSs	Average Zeta potential (ζ) mV	Electrophoretic mobility (μ)	Average DLS size d (nm)
MoS_2 (non-irradiated)	-32.1 ± 1.5	-2.2 ± 0.4	190 ± 10 nm
MoS_2 (Irradiated)	-29.8 ± 1.6	-2.3 ± 0.6	220 ± 10 nm

Table S1: Represents the average linear size d (nm) of the nanosheets as estimated by DLS measurements and the average ζ-potential value of the NSs for treated and untreated cases. The analysed NS sample was fabricated by centrifuging at 1000 g.

SEM-EDS for morphological and semi-quantitative chemical Analysis

The morphological properties of MoS_2 flakes were examined in pristine and Au-coated conditions by using a field emission gun scanning electron microscope Zeiss Leo 1530 model (ENEA, Centro Ricerche Casaccia laboratory) working at low voltage (i.e., 8kV). Data image processing was done using Software controller FeSEM: SmartSem. A Zeiss Merlin VP compact equipped with charge compensation system and with Oxford Instruments Microanalysis both EDS X-max 50 and WDS Wave, (DiSTAR laboratory, Università degli Studi di Napoli Federico II), was used for semi quantitative chemical analyses of the samples. The Electron Microscope Field emission Zeiss Merlin VP Compact with camera Gemini II is composed by three secondary electron detectors SE2 (Classic Detector), VPSE (Variable Pressure) and InlensDuo (Low Voltage) and by two backscattered electron detectors AsB and InlensDuo. Data processing was done using INCA (EDS e WDS) e Aztec (EDS). The samples were placed on a glass support by appropriate droplet drop casting; then, they were metalized with graphite by using a sputter coater. The semi-quantitative chemical analyzes were carried out by imposing the closure at 100wt%, for an immediate reading of the data. For each sample 20 analysis points were performed.

Raman Micro-spectroscopy of 2D-MoS_2

In our experiment, Raman spectra were acquired by using a commercial micro-Raman system (WiTec, Alpha 300) endowed with a Raman probe at λ_{exc} = 532 nm. A typical Raman spectrum of the of the produced nanoflakes exhibits the two characteristic bands, $E\frac{1}{2g}$ and A_{1g},

corresponding to in-plane and out-of-plane vibrational modes[3,4] According to literature[5–7] the frequency shift $\Delta\boldsymbol{\vartheta}_{MoS2} = \boldsymbol{\vartheta}_{A_{1g}} - \boldsymbol{\vartheta}^{1}_{2g}$ between these modes can be used to identify the number of layers *n* in 2D-MoS_2. Therefore, to get this parameter for the flakes produced by our protocol, we performed a Raman analysis on exfoliated MoS_2 deposited on a coverslip. In particular, 50 µL of the nanoflakes suspension were dropped on a glass coverslip and allowed to dry at room temperature.

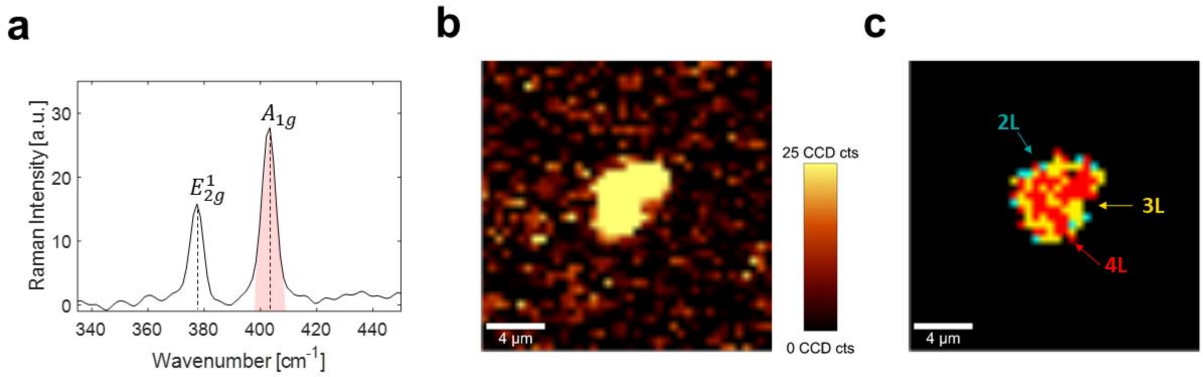

Figure S2: a) Typical Raman spectrum of exfoliated 2D-MoS_2, showing the two characteristic peaks of E^1_{2g} and A_{1g} modes at about 380 cm^{-1} and 403 $^{-1}$, respectively; b) mapping of the band intensity distribution of the A_{1g} spectral peak highlighted in red in a), obtained in a raster scanning of a 2D-MoS_2 agglomerate in a 20 µm × 20 µm region. Acquisition parameters were 7 mW laser beam power and 0.5 s acquisition time, respectively, while the step size was 500 nm. c) Map reporting the number of layers for the agglomerate of flakes shown in b)

In Figure S2 we show a typical Raman spectrum of exfoliated 2D-MoS_2, showing the two characteristic peaks of E^1_{2g} and A_{1g} modes at about 380 cm^{-1} and 403 $^{-1}$, respectively (a); a mapping in b of the band intensity distribution of the A_{1g}spectral peak highlighted in red in a, obtained by a raster scanning of a 2D-MoS_2 agglomerate in a 20 µm × 20 µm region. The Raman map in b clearly reveals the presence of a nanoflake agglomerate in its central region. In c a map is shown reporting the number of layers for the agglomerate of flakes shown in b, estimated from the difference of the two characteristic peaks positions (2L: 23.4 cm^{-1}, 3L: 24.6 cm^{-1}, 4L: 25.9 cm^{-1}) [46]

Atomic Force Microscopy (AFM)

To analyse the nanoflakes morphology, Atomic Force Microscopy (AFM) was also employed. At this purpose, 50 μL of 2D-MoS_2 suspension was dropped onto a Si/SiO_2 substrate heated at ~ 100 °C and allowed to evaporate. This procedure strongly reduced 2D-MoS_2 aggregation. AFM measurements were performed in AC mode, so that in each scan both phase and morphological (height) maps were acquired.

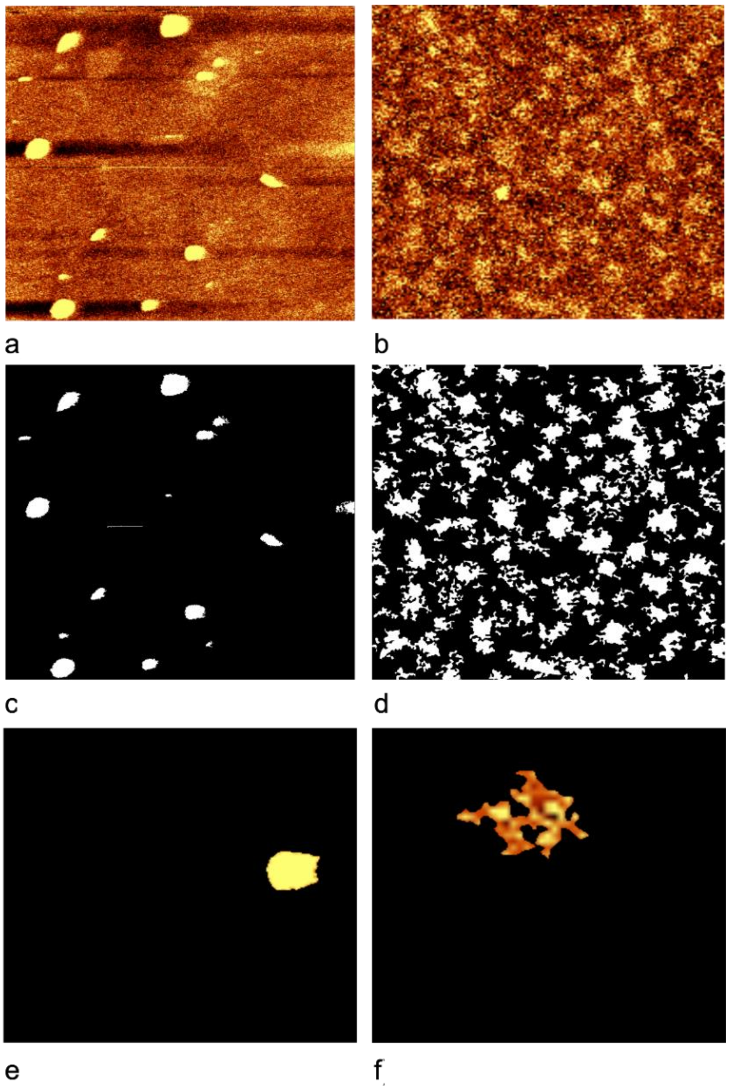

Figure S3. a AFM image of treated 2D MoS_2 nanoflakes b AFM image of untreated 2D MoS_2 nanoflakes c - d segmentation masks applied as pre-processed step to select treated and untreated MoS_2 nanoflakes; e - f typical segmented treated and untreated MoS_2 nanoflakes. "

ImageJ software was used for a quantitative analysis of the AFM images in terms of area, perimeter, circularity, and roughness. The algorithm operates a specific background subtraction for the acquired images, creating a mask for segmenting the nanoflakes in the field of view. Therefore, 2D-MoS_2 were counted and analysed. In particular, the following values were obtained: *i)* the area A corresponding to the number of pixels assigned to each nanoflake, *ii)* the perimeter P given by the length of its boundaries, and *iii)* the solidity S , a dimensionless quantity given by the following formula:

$$S = \frac{A}{A_c} \tag{1}$$

where A_c is the convex area associated with the convex hull in which the nanoflake can be inscribed. On the basis of the previous parameters, the form factor was also estimated F

$$F = \frac{4 \cdot \pi \cdot A}{P \cdot P} \tag{2}$$

.

Finally, 2D-MoS_2 roughness was calculated, by taking advantage of a plug-in written for ImageJ by Chinga et al. The roughness was estimated starting from the mean deviation (Ra) of pixels from available topographical images.

X-ray Photoelectron Spectroscopy (XPS)

X-Ray Photoelectron spectra were recorded on a XPS Versa Probe II (PHI, Chanassen US) by large area analysis mode where the monochromated Al anodic beam of 100 µm, at 100 W power, normal to the surface, is rastered over an area of 1400 µm × 300 µm with the analyser at 45° to the sample surface. Survey spectra were acquired with an accumulation time at least of 20 minutes at high pass energy (187 KeV) while high resolution spectra of the elements of interests were acquired at 11.7 KeV.

Spectra were analysed by Multipack (PHI, Chanassen US) software and all the peaks were referenced to the adventitious carbon peaks C 1s at 284.8 eV binding energy. Samples were prepared by dropping a suspension of the aqueous suspensions of MoS_2 on a flat surface of silicon and the liquid evaporated in a vacuum chamber. As a control the suspending aqueous media were also analysed to exclude potential contaminations (data not shown).

Scanning Electron Microscopy (SEM)

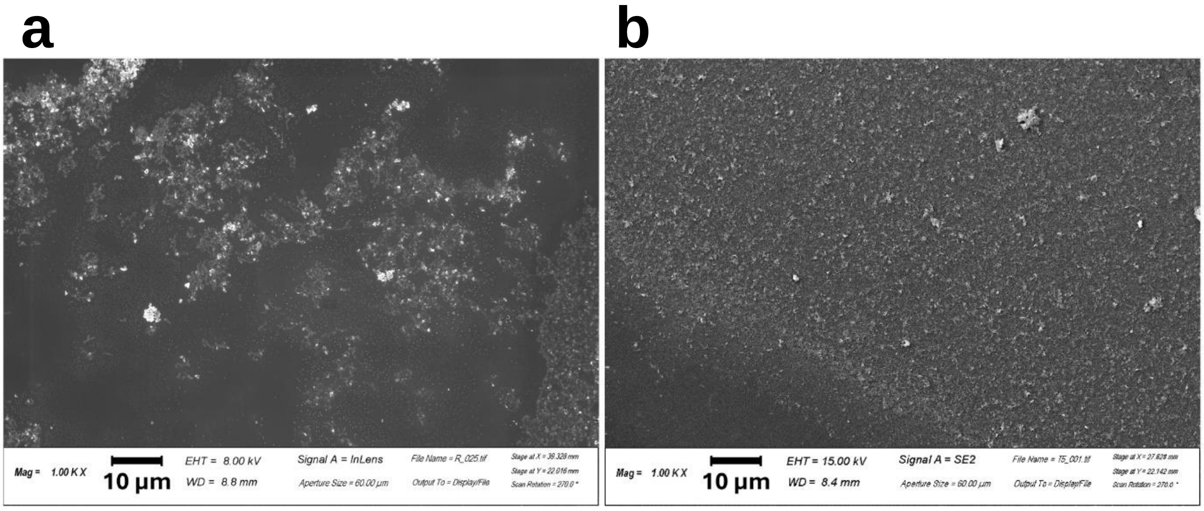

Figure S4: Distribution of MoS_2 nanoparticles in non-irradiated but exfoliated MoS_2 NSs and in the irradiated MoS_2 samples b, sometimes large aggregates are visible.

	**Mean Range (mm)**	**Maximum range (mm)**
**Water**	2.24	8.92
**Glass**	0.83	3.30

Table S2: Mean and maximum range of positrons from ^{68}Ga in water and glass

	**Treated**		**Untreated**	
**Parameters**	**Solidity**	**Form Factor**	**Solidity**	**Form Factor**
**Mean ± std**	0.8±0.1	0.5±0.2	0.6±0.1	0.3±0.2
**Min - Max**	0.4-1	0.1-0.9	0.4-0.9	0.1-0.9

Table S3: Statistical analysis of Solidity and Form factor in treated and untreated cases.

	**Treated - Ra value in nm**	**Untreated - Ra value in nm**
**Mean**	0.11 ± 0.47	0.15 ± 1.4
**Min - Max**	0.09 - 9.01	0.01-1.4

Table S4: Statistical analysis of Ra (peak-to-valley) roughness parameters in treated and untreated cases.

	**Untreated**	**Treated (not altered)**	**Treated (altered)**
	**Mean (wt%, weight percent)**		
**S**	40.1±0.6	40.1±0.3	35±2
**Mo**	59.9±0.6	59.9±0.3	65±2
**Tot**	100.00	100.00	100.00
	**APFU (Atoms Per Formula Unit)**		
**S**	1.25±0.02	1.2±0.3	1.1±0.1
**Mo**	0.62±0.01	0.6±0.3	0.68±0.02
**S/Mo**	2.01±0.03	2.0±0.6	1.60±0.1
	**Formula**		
	MoS_2	MoS_2	$MoS_{1.60}$

Table S5: Stoichiometric effects of ionizing radiation on a statistical sample of 20 analysed MoS_2 NSs.

	Spectral window	Pristine		MoS_2 Sonicated		2D NSs Sonicated Centrifuged		2D NSs – TS Sonicated Centrifuged Irradiated	
		Pos	***% at***	***Pos***	***% at***	***Pos***	***% at***	***Pos***	***% at***
S 2p (MoS_2)	159 – 173 eV	162.29	*66.67*	162.30	*61.75*	162.29	*55.31*	162.28	*60.15*
		163.47	*33.33*	163.48	*30.88*	163.47	*27.66*	163.46	*30.08*
S 2p (Sulfate)		-	-	168.07	*4.91*	168.25	*11.35*	167.86	*6.51*
		-	-	169.25	*2.46*	169.43	*5.68*	169.04	*3.26*
S 2s (MoS_2)	225 – 239 eV	226.83	*13.98*	226.74	*13.50*	226.77	*13.51*	227.64	*10.10*
S 2s (organosulfur)				228.47	*3.02*	228.48	*3.63*	-	-
Mo IV 5/2 - $Mo^{IV}S_yO_x$		229.51	*48.48*	229.42	*45.93*	229.46	*43.28*	229.48	*24.60*
Mo IV 3/2 - $Mo^{IV}S_yO_x$		232.64	*32.48*	232.55	*30.77*	232.59	*28.99*	232.61	*16.48*
Mo^V - $Mo^VS_yO_x$		-	-	-	-	-	-	230.45	*25.37*
Mo V 3/2 - $Mo^VS_yO_x$		-	-	-	-	-	-	233.58	*17.00*
S 2s (Sulfate)		-	-	-	-	-	-	233.45	*3.26*
Mo VI 5/2 - $Mo^{VI}S_yO_x$		232.87	*2.67*	232.48	*4.06*	232.66	*6.33*	233.46	*1.92*
Mo VI 3/2 - $Mo^{VI}S_yO_x$		236.00	*1.79*	235.61	*2.72*	235.79	*4.24*	236.59	*1.28*

Table S6: Spectral position of energy peaks in the XPS spectra and quantitative surface composition of samples after deconvolution of S and Mo envelops.

	Relative Sensitivity Factor (RSF) Corrected	Pristine	MoS_2 Sonicated	2D NSs	2D NSs -TS
C 1s	*8.944*	7.88	13.69	14.12	12.76
**O 1s**	*21.107*	3.75	12.63	25.42	12.02
**S 2p**	*7.888*	28.33	24.38	20.88	24.38
**Mo 3d**	*39.479*	60.05	49.30	39.58	50.84

Table S7: shows the position and quantitative surface composition of samples after deconvolution of S and Mo envelops. It clearly shows a comparison between the 2D NSs and the treated samples exhibiting the shift in the BE peaks after the irradiation procedure.

References

1. Singh, M. *et al.* Combating Actions of Green 2D-Materials on Gram Positive and Negative Bacteria and Enveloped Viruses. *Front Bioeng Biotechnol* **8**, (2020).

2. Kaur, J. *et al.* Biological interactions of biocompatible and water-dispersed MoS2 nanosheets with bacteria and human cells. *Sci Rep* **8**, (2018).

3. Li, H. *et al.* From bulk to monolayer MoS 2: Evolution of Raman scattering. *Adv Funct Mater* **22**, 1385–1390 (2012).

4. Rusciano, G. *et al.* Single-Cell Photothermal Analysis Induced by MoS2 Nanoparticles by Raman Spectroscopy. *Front Bioeng Biotechnol* **10**, (2022).

5. Berkdemir, A. *et al.* Identification of individual and few layers of WS2 using Raman Spectroscopy. *Sci Rep* **3**, (2013).

6. Mignuzzi, S. *et al.* Effect of disorder on Raman scattering of single-layer Mo S2. *Phys Rev B Condens Matter Mater Phys* **91**, (2015).

7. Peimyoo, N. *et al.* Thermal conductivity determination of suspended mono- and bilayer WS2 by Raman spectroscopy. *Nano Res* **8**, 1210–1221 (2015).

8. Kaur, J. *et al.* Green synthesis of luminescent and defect-free bio-nanosheets of MoS2: Interfacing twodimensional crystals with hydrophobins. *RSC Adv* **7**, 22400–22408 (2017).
